# Does Arterial Hypertension Affect Plasma Levels of Matrix Metalloproteinases and Their Tissue Inhibitors in Patients with Stable Coronary Artery Disease? A Preliminary Study

**DOI:** 10.1155/2019/6921315

**Published:** 2019-11-04

**Authors:** Wiktor Kuliczkowski, Marta Banaszkiewicz, Andrzej Mysiak, Grzegorz Makaś, Iwona Bil-Lula

**Affiliations:** ^1^Department and Clinic of Cardiology, Wroclaw Medical University, 50-367 Wroclaw, Poland; ^2^Division of Clinical Chemistry, Department of Medical Laboratory Diagnostics, Faculty of Pharmacy with Division of Laboratory Diagnostics, Wroclaw Medical University, 50-367 Wroclaw, Poland; ^3^Department of Cardiology, Provincial Hospital Center of the Jeleniogorska Valley, 58-501 Jelenia Gora, Poland

## Abstract

**Background:**

Arterial hypertension (HT) is a serious and prevalent epidemiological factor in the development of coronary artery disease (CAD). Metalloproteinases (MMPs), especially MMP-2 and MMP-9, and their natural endogenous tissue inhibitors (TIMPs) are involved in the pathogenesis of HT and its complications. MMPs are also involved in the development of diabetes (DM), a risk factor for CAD. The aim of the study was to explore the influence of CAD, HT, and DM on changes in plasma levels of MMP-2 and MMP-9 and their inhibitor TIMP-4.

**Methods and Results:**

The study involved 70 patients with stable CAD admitted for coronary angiography and 15 healthy subjects. Whole blood samples were collected prior to angiography. MMP-2, MMP-9, and TIMP-4 levels in plasma were estimated using ELISA tests. CAD patients showed a significantly increased level of TIMP-4 and decreased level of MMP-2 in comparison to healthy controls (*p*=0.011 and *p*=0.037, respectively). Concentration of MMP-2, MMP-9, and TIMP-4 did not differ in the group with and without hypertension. Patients with DM presented higher MMP-2 level than patients without DM (*p* < 0.001). Multiple regression analysis of the influence of independent variables such as CAD stage, DM, and HT on MMP-2, MMP-9, and TIMP-4 showed that only DM was independently associated with a higher level of MMP-2 (*β* = 0.42, *R*^2^ = 0.17, *p* < 0.001).

**Conclusion:**

Data showed that patients with CAD presented higher TIMP-4 and lower MMP-2 concentration regardless of HT and DM. HT had no effect on MMP-2, MMP-9, and TIMP-4 levels in serum. DM was independently associated with higher MMP-2 concentration; however, co-occurrence of CAD and DM was associated with the balance in the MMP-2 level. Concentration of MMP-9 did not change significantly in any of the analysed groups.

## 1. Introduction

Coronary heart disease is a condition of myocardial ischemia associated with changes in coronary arteries. Chronic angina pectoris, cardiac X syndrome, and angina associated with muscular bridges over the coronary arteries are classified as stable coronary artery disease (CAD). The most common cause of ischemic heart disease is coronary atherosclerosis, in which smooth myocytes that capture lipids grow in the wall of the coronary artery [1]. Endothelium is damaged thus causing the appearance of small wall clots with platelets, secreting platelet-derived growth factor (PDGF). PDGF stimulates the proliferation of myocytes and activates them for the synthesis of collagen. The turnover of collagen is regulated by extracellular matrix metalloproteinases (MMPs) [2].

MMPs constitute a family of endoproteases that play a major role in extracellular matrix (ECM) homeostasis [3]. They degrade the protein components of ECM, thus causing its regeneration and remodeling. In this way, the proper structure of the matrix and basal membrane are preserved in both physiological and pathological processes [4, 5]. Hereby, the proliferation and migration of myocytes are facilitated by MMPs [6]. As collagen appears, fibrous plaque turns into white and lymphocytes accumulate on its periphery. Atherosclerotic plaques undergo further changes, calcification or decay [7].

Coronary artery disease is accompanied by many diseases, including arterial hypertension (HT) [7, 8]. Increased blood pressure in the circulatory system initiates inflammation in the arterial wall [5]. It is well established that many MMPs and natural endogenous tissue inhibitors (TIMPs) are involved in the pathogenesis of hypertension and its further complications [9–12]. At the incipient stages of hypertension, metalloproteinases have a protective function, allowing the blood vessels to adapt to the new conditions of increased blood pressure. Over time, the progress of blood vessel remodeling leads to the development of its pathology [13, 14].

Hypertension activates MMP-2 through mechanical and oxidative stress [1, 15]. MMP-2 leads to the proteolysis of ECM components and participates in the transformation and migration of smooth muscle cells of blood vessels [15]. Reconstruction of blood vessels as a result of hypertension may be the first step in the development of atherosclerosis, stroke, and renal and heart failure [16]. For this reason, MMPs are considered as a therapeutic target, since the inhibition of their activity may reduce hypertension, prevent complications, and prevent the occurrence of fatal cardiovascular events [6, 14].

MMPs are also involved in the development of type 1 and type 2 diabetes (DM) [17]. In more than half of patients with type 2 diabetes, coronary artery disease develops [18, 19]. Diabetes and hypertension are the risk factors for cardiovascular diseases [20]. In the course of diabetes, hyperglycaemia induces the production of proinflammatory cytokines, leading to proliferation and migration of vascular smooth muscle cells (VSMC), the subsequent transformation of the vascular basal membrane, and the formation and development of atherosclerotic plaques [21]. High glucose levels cause an increased production of free radicals and also inhibit estrogen antiproliferative activity [22]. Hyperglycaemia also causes an increase in the expression and activity of MMP-1 and MMP-2 in endothelial cells and MMP-9 in macrophages, which are all involved in atherosclerotic plaque development [23]. MMP-2 and MMP-9 are firstly synthesized in atherosclerotic lesions and then activated by reactive oxygen species (ROS) [24]. In turn, increased concentration of MMPs in atherosclerotic plaque correlates with its rupture and development of unstable angina pectoris [25].

The aim of the study was to explore the influence of CAD, HT, and DM on changes in plasma levels of MMP-2 and MMP-9 and their inhibitor TIMP-4. TIMP-4 and MMPs form complexes in a 1 : 1 ratio in which TIMP-4 blocks substrate access to the MMP catalytic site [15]. We suggest that the increased production of MMPs, due to the mechanical stress induced by hypertension, should be accompanied by an increased expression of TIMP-4 [26]. This study may serve as a preliminary test to determine whether TIMP inhibition of MMPs may help with the prevention of cardiovascular complications resulting from the coexistence of hypertension, diabetes, and coronary artery disease.

## 2. Materials and Methods

### 2.1. Study Group and Clinical Material

This study consisted of seventy patients diagnosed with coronary artery disease, invited by the Department and Clinic of Cardiology, Wroclaw Medical University. Patient characteristics are presented in Table 1. Fifteen healthy volunteers, adjusted with age and sex, were also recruited and served as healthy controls. In the study group, there were fifty-seven patients with hypertension, out of whom thirty-two suffered from diabetes. Each participant was precisely informed about the purpose and the principles of the study and signed consent for blood sample collection. 10 ml of citrate anticoagulated whole blood (1 + 9, v : v) was obtained from the subjects. The study was approved by the local Ethics Committee of Medical University of Silesia (NN-6501-25/I/07).

### 2.2. Criteria for Classification

The study employed inclusion criteria such as stable coronary artery disease, hypertension, and diabetes mellitus. Exclusion criteria included percutaneous coronary intervention (PCI) and coronary artery by-pass grafting (CABG) up to 3 months before entering the study, current bleeding, anaemia, platelets count in whole blood below 150 000/mm^3^ or above 450 000/mm^3^, and intake of antiplatelet drugs other than aspirin during two weeks before study inclusion.

### 2.3. Concentrations of MMP-2, MMP-9, and TIMP-4 in Plasma

Samples of sodium citrate anticoagulated blood collected on ice were centrifuged (1000 ×*g*, 20 min, 4°C) instantly after acquisition. Separated plasma was used for the measurement of MMPs and TIMP-4 concentrations. ELISA tests for MMP-2 (Total MMP-2 Quantikine ELISA), MMP-9 (Human MMP-9 Quantikine ELISA), and TIMP-4 (Human TIMP-4 Quantikine ELISA) (all from R&D, USA) were performed. Total MMP-2 Quantikine ELISA assay recognized recombinant MMP-2, natural human, mouse, rat, porcine, and canine active, pro-, as well as TIMP-complexed MMP-2. Human MMP-9 Quantikine ELISA test measured natural and recombinant 92 kDa pro-MMP-9 and 82 kDa active MMP-9. It was not designed to measure the 65 kDa form of MMP-9. Human TIMP-4 Quantikine ELISA recognized natural and recombinant human TIMP-4. MMP-2/MMP-9/TIMP-4 was immobilized with a monoclonal antibody specific to adequate protein and was detected using anti-MMP-2, anti-MMP-9, or anti-TIMP-4 polyclonal antibody conjugated to horseradish peroxidase (HRP). Then, TMB substrate solution was added to trigger the reaction. The limit of detection was estimated averagely at 0.033 ng/mL for MMP-2, less than 0.156 ng/mL for MMP-9, and 4.91 pg/mL for TIMP-4.

### 2.4. Statistics

Data analysis was performed using Statistica 12 software (StatSoft, USA). Results were presented either as mean ± SEM or median (interquartile range). Right-skewed distribution data, which fit the remaining criteria for the normal distribution, were logarithmically transformed and analysed by appropriate tests: Shapiro-one-way or two-way ANOVA, Mann–Whitney *U* test, and unpaired *t*-test. The confirmation of compared group homogeneity was done with *χ*^2^ test with Yates' correction. As a statistically significant difference, *p* < 0.05 was accepted. Graphs were made by GraphPad Prism v. 5.

## 3. Results

### 3.1. Plasma Levels of MMP-2, MMP-9, and TIMP-4 in CAD Patients

**Figure 1:**
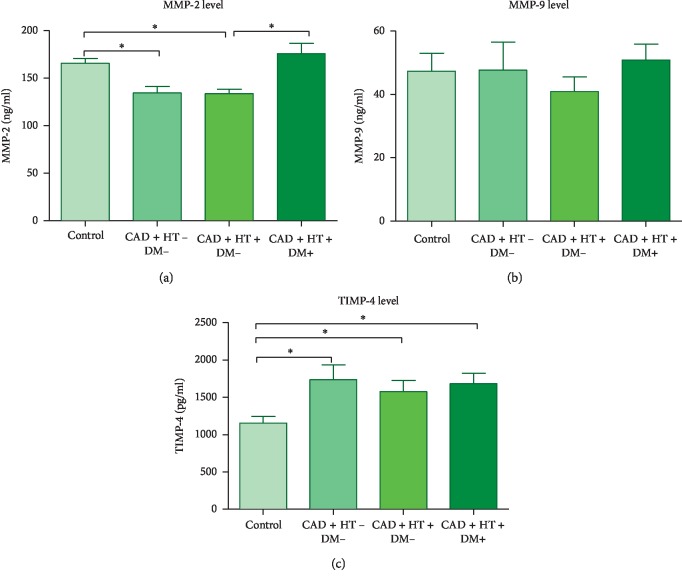
Levels of MMP-2 (a), MMP-9 (b), and TIMP-4 (c) in all analysed groups (^*∗*^ − *p* < 0.05). CAD + HT − DM−: group of patients suffering from coronary artery disease only; CAD + HT + DM−: group of patients suffering from coronary artery disease and hypertension without diabetes; CAD + HT + DM + : group of patients suffering from coronary artery disease, hypertension, and diabetes; Control: control group; MMP-2: matrix metalloproteinase-2; MMP-9: matrix metalloproteinase-9; TIMP-4: tissue inhibitor of metalloproteinases 4. Statistical tests used: Mann–Whitney *U* test and unpaired *t*-test.

A comparison of the whole study group (*n* = 70) with respect to the control group (*n* = 15) showed significantly increased TIMP-4 and decreased MMP-2 concentrations in CAD patients (*p*=0.011 for TIMP-4; *p*=0.037 for MMP-2), whereas there were no differences in MMP-9 concentration between groups (see Table 2). To exclude the influence of HT and DM, the analysis of CAD without comorbidities was performed. CAD + HT-DM− group (*n* = 11) was compared to the control. Data showed increased TIMP-4 and decreased MMP-2 levels (*p*=0.008 and *p* < 0.001, respectively) (see Figure 1). Comparison of patients from CCSII (*n* = 62) and CCSIII (*n* = 8) groups showed no significant differences in MMP-2, MMP-9, and TIMP-4 levels (see Table 2).

### 3.2. Influence of HT on Plasma Levels of MMP-2, MMP-9, and TIMP-4

Next, it was examined whether the coexistence of CAD and HT influenced the analysed parameters. CAD + HT + DM− group in relation to the healthy control showed the same effect as before, increased TIMP-4 and decreased MMP-2 level in the study group (*p*=0.038 and *p* < 0.001, respectively). However, HT alone did not affect MMP-2, MMP-9, and TIMP-4 levels (see Table 3; Figure 1).

### 3.3. Influence of DM on Plasma Levels of MMP-2, MMP-9, and TIMP-4

Due to the low number of subjects in CAD + HT − DM+ group, the analysis could not be performed; we made a comparison of CAD + HT + DM+ group to the control, as it was proved before that HT did not affect the analysed parameters. This comparison showed no differences (Table 4). The comparison of CAD + HT + DM +  group (*n* = 32) to CAD + HT + DM− group (*n* = 25) showed increased MMP-2 level in the first group (*p*=0.0025) (see Table 4; Figure 1).

### 3.4. Multiple Regression Analysis

Multiple regression analysis of the influence of independent variables such as CAD stage, DM, and HT on plasma levels of MMP-2, MMP-9, and TIMP-4 showed that only DM was independently associated with a higher level of MMP-2 (*β* = 0.42, *R*^2^ = 0.17, *p* < 0.001) (see Table 5).

## 4. Discussion

MMPs are synthesized and secreted by many cell types: fibroblasts, vascular endothelial cells, cardiac muscle cells, macrophages, and blood cells such as lymphocytes, neutrophils, or eosinophils. Their substrates comprise primarily ECM components such as collagen, elastin, laminin, proteoglycan, denatured collagen (gelatin), and others [4, 27]. MMPs degrade ECM components, affect remodeling of the vascular tissue, and contribute to the stiffness of vascular walls [28, 29]. The levels of circulating MMPs can increase in cardiovascular diseases. MMP-2 and MMP-9 are released from inflammatory cells, which means that their level in the circulation increases during inflammation [6, 29]. These two MMPs were also found in VSMC, in which both indicate that they can affect the development of atherosclerosis and atherosclerotic plaque rupture [26, 30]. The balance between MMPs and TIMPs is very important; it gets unstable in chronic remodeling processes, e.g., as a result of myocardial infarction and heart failure [31]. TIMP-4 is known to slow down the process of activation of MMP-2 [32]. It is also called the cardiac inhibitor of MMPs as it plays an important homeostatic role in myocardium. TIMP-4 protects the heart against damage by inhibition of MMP-2 and MMP-9 proteins [26]. Most studies that address this issue speak of reduced levels of TIMPs and increased levels of MMPs in the heart [3]. Our research shows that the TIMP-4 level was significantly elevated in subjects with CAD, while the level of MMP-2 was reduced. These results were not associated with the presence or absence of comorbid diseases such as HT or DM. It may suggest that in stable CAD, regardless of the stage of this disease, there were attempts to restore the balance—whereby the initially increased level of MMP-2 was then lowered by TIMP-4, the level of which also increased—a compensatory feedback mechanism occurred [31].

Hypertension is also closely related to vessel remodeling and rearrangement of many components of the vascular wall, including ECM proteins [4, 31, 33]. In the early and moderate stages of hypertension, one may observe an eutrophic vessel remodeling, in which VSMC reorganize around the lumen of the vessel and change it - an increase in MMPs activity may be helpful as it aids vessels to adapt to the increased blood pressure [14, 15, 31, 34]. However, when hypertension persists and turns severe, the eutrophic remodeling changes into hypertrophic. It occurs mainly in large vessels such as the aorta and leads to a significant increase in the thickness of the wall and reduction in the lumen of the vessel [16]. This is associated with increased collagen deposition together with elastin fragmentation and significantly contributes to the stiffness of the vessel [35]. During the reconstruction, new elastin and collagen are resynthesized by VSMC, which in turn causes an exacerbation of hypertension [5, 11, 15]. Increasing vascular stiffness is considered as a determinant of cardiovascular risk [35]. Hypertension also contributes to the activation of MMP-2 and vascular remodeling through the mechanical stress induction. Mechanical stress increases the level of MMP-2 in VSMC by activating the platelet-derived growth factor mechanoreceptor (PDGF-R) and protein kinase B signaling/Akt pathway [15].

Oxidative stress is also an important factor in regulating the expression of MMP-2. The increased production of ROS in vessels contributes to the activation of MMP-2, thereby inducing the development of hypertension and other chronic cardiovascular alterations [15, 16]. In our study, no significant relationship was found between HT and MMP-2, MMP-9, and TIMP-4 concentrations. There are some previous works showing the relationship, but results provided by other researchers are inconsistent. Trojanek et al. (2015) studied MMP-2 and MMP-9 levels in people with hypertension in relation to a healthy control group. A significantly higher level of MMP-9 was observed in people with hypertension, especially in its early phase, and no differences in the remaining subjects. MMP-9 degrades the collagen type IV, V, VII, and X, gelatin, elastin, and other ECM proteins, which indicates its important role in the metabolism of the basal vascular membrane. It is secreted by macrophages and activated in the vascular wall, participating in processes of degeneration, hyperplasia, and narrowing of their lumen, and its activity increases under the conditions of inflammation. As it is known, hypertension is associated with the development of inflammation, vascular remodeling, and the rearrangement of many components of the vascular wall [4]. The authors noticed a significant increase only in the level of MMP-9, while in MMP-2 and TIMP-1, although also elevated, did not demonstrate a statistically significant rise [4]. Marchesi et al. (2011) showed that concentrations of MMP-2, MMP-9, and TIMP-1 were higher in hypertensive patients than those in healthy subjects; these MMPs and TIMP-1 were considered as the best cardiovascular markers for remodeling under hypertension [8]. The same results were obtained by Raffetto and Khalil [31]. Castreo et al. examined hypertensive rats to the healthy control group - they had significantly elevated levels of MMP-2 and MMP-9, but not TIMP [33]. It was established that MMPs were elevated due to increased oxidative stress and the action of ROS, which, as mentioned before, also affect the increased secretion of MMPs [15, 16, 33]. The increased level of MMPs resulted as well from the fact that hypertension in these rats was already chronic and hypertrophic vascular remodeling occurred [33]. The inconsistency of results could be caused, e.g., by the differences in the stage of particular disease, as well as the type of additional complications in patients qualified for research, the diversity of conditions in which research was conducted, population differences (age, sex, and biogeographic location), as well as the statistical power of results [4]. In our group, the diseases overlapped, which made it difficult to relate our results to the results obtained by other groups of researchers. Other authors tested the effect of one factor such as CAD, HT, or DM, while in our study, most patients suffered from CAD and other comorbidities such as HT or DM. In this context, it seems obvious that the topic requires further research and the extension of research to a larger number of patients.

The influence of DM on the level of MMPs in plasma cannot be neglected either. High glucose levels in people with diabetes cause dysregulation of the MMP/TIMP pathway, which in consequence results in imbalance between the synthesis and degradation of the ECM [10]. Diabetes is a chronic inflammatory disease in which the induction of proinflammatory factors leads to the accumulation of monocytes, macrophages, and granulocytes, being a rich source of MMPs [18]. There is also a significant increase in the development of diseases such as CAD, myocardial infarction, and congestive heart failure in patients with diabetes [20, 25, 36]. Our results showed that the level of MMP-2 was significantly elevated in the presence of diabetes. We also noticed that DM and CAD had a reverse effect on the MMP-2 level, as DM caused increase while CAD was associated with decrease in MMP-2 concentration. There was no significant increase in MMP-9 and TIMP-4 due to the presence of diabetes. However, Derosa et al. showed that diabetic patients had significantly higher levels of MMP-2 and MMP-9, as well as TIMP-1 and TIMP-2, in relation to the healthy control group [6]. Similar results were obtained by Signorelli et al. —MMP-2 and MMP-9 were also elevated in diabetic patients relative to the control, but TIMP concentration has not been tested [24]. Naduk-Kik and Hrabec showed that MMP-9 was positively correlated with the progression of diabetes [21], just like MMP-2 with diabetes occurrence in current research. Feng and Jin-De, in turn, studied the correlation between DM and the level of MMP-2 in rats. They observed increased glucose level in rats causing an increase in the expression of MMP-2 as well. They showed that lowering glucose levels not only reduced the symptoms associated with hyperglycaemia but also reduced the risk of cardiovascular disease and the risk of death [22].

Hypertension that occurs with diabetes may enhance the complications resulting from the presence of this disease, particularly cardiovascular diseases and nephropathy [36]. Kostov et al. conducted a study in which patients with type 2 diabetes were divided into prehypertensive and hypertensive groups. MMP-2 concentration was measured. In comparison of the prehypertensive group to the control group, it was significantly increased in the first group. There were no statistically significant differences between the prehypertensive and hypertensive groups. However, it was noticeable that in the hypertensive group, the concentration of MMP-2 was lower than that in the prehypertensive group, while it was higher than that in the control. This indicates that the balance between the synthesis and degradation of ECM components in the vessel walls changes dynamically as the disease develops. It most likely follows that the expression of MMP-2 is induced at the beginning of hypertension process; therefore, its increased levels mainly reflect early changes in ECM, provided that there are no other vascular complications. There were no significant differences in MMP concentrations in the results obtained by us in people with hypertension, probably due to the fact of coexistence of other coronary risk factors—CAD that influenced MMP/TIMP concentrations.

## 5. Conclusions

In conclusion, our research showed that there was an elevated level of TIMP-4 and a decreased level of MMP-2 in stable CAD. These data may suggest that in stable CAD, the compensatory feedback mechanism was observed, so the initially increased MMP-2 level was then lowered by TIMP-4. An additional risk factor such as hypertension did not cause significant changes in the levels of MMP-2, MMP-9, and TIMP-4. Diabetes was independently correlated with an increased level of MMP-2; however, CAD and DM occurring together were associated with the balance in the MMP-2 level. Concentration of MMP-9 did not change significantly in any of the analysed groups. In this context, it seems obvious that MMP-2, MMP-9, and TIMP-4 balance was highly dependent on the clinical stage of the disease and other coexisting risk factors. For this reason, this issue requires further research due to the overlapping of diseases, as well as increase in the number of examined patients.

## Figures and Tables

**Table 1:** Characteristics of the study population.

Clinical parameter	CAD group		Statistical significance: arterial hypertension vs no arterial hypertension group
	Arterial hypertension	No arterial hypertension	
Total number of patients (%)	57	13	
Age (years), mean ± S.E.M.	62.4 ± 8.3	57.3 ± 8.9	ns
Sex			ns
Men	44	11	
Women	13	2	
Clinical characteristics			
Coronary artery disease			ns
CCS II^*∗*^	50	12	
CCS III^*∗*^	7	1	
Diabetes mellitus	32	2	*p*=0.0189^*∗∗*^
Current tobacco use	19	6	ns
History of myocardial infarction	29	5	ns
History of PCI/CABG >3 months before study	PCI 22 CABG 8	PCI 6 CABG 2	ns
History of stroke or TIA >3 months before study	4	1	ns
Kidney insufficiency (GFR <60 ml/min/m^2^)	11	4	ns
Hypercholesterolemia	55	10	ns
HbA1C, mean ± SD	6.5 ± 3.4	5.9 ± 2.8	ns
Drug administration			
Beta-blocker	57	10	ns
Calcium channel blockers	42	2	ns
ACE-I	55	6	ns
ARB	13	2	ns
Statins	57	13	ns
Oral antidiabetic drugs	28	2	ns
Insulin	20	1	ns

ACE-I: angiotensin-converting enzyme inhibitor; ARB: angiotensin receptor blocker; CABG: coronary artery by-pass grafting; CAD: coronary artery disease; GFR: glomerular filtration rate; HbA1C: glycated hemoglobin A1C; NA: not analysed; NS: statistically not significant; PCI: percutaneous coronary intervention; SD: standard deviation; TIA: transient ischemic attack. ^*∗*^CCS: Canadian Cardiovascular Society classification of coronary artery disease clinical status. ^*∗∗*^*χ*^2^ Yates.

**Table 2:** Influence of CAD on plasma levels of MMP-2, MMP-9, and TIMP-4.

	CAD	Control	*p* value	CAD + HT − DM−	Control	*p* value	CCSII	CCSIII	*p* value
MMP-2 (ng/ml)	154.5 ± 51.0	165.7 ± 19.1	**0.037**	134.5 ± 23.2	165.7 ± 19.1	**<0.001**	154.0 ± 51.6	158.8 ± 49.6	0.77
MMP-9 (ng/ml)	46.4 ± 26.4	47.2 ± 22.3	0.91	47.6 ± 29.4	47.2 ± 22.3	0.97	45.5 ± 25.4	54.2 ± 34.8	0.71
TIMP-4 (pg/ml)	1621.9 ± 757.4	1153.2 ± 339.9	**0.011**	1733.0 ± 665.4	1153.2 ± 339.9	**0.008**	1631.8 ± 788.0	1546.1 ± 487.4	0.85

CAD: coronary artery disease; CAD + HT − DM−: group of patients suffering from coronary artery disease only; CCSII, CCSIII: Canadian Cardiac Society classification of coronary artery disease clinical status: stage II and stage III, respectively; MMP-2: matrix metalloproteinase-2; MMP-9: matrix metalloproteinase-9; TIMP-4: tissue inhibitor of metalloproteinases 4. Mean ± SEM. Statistical tests used: Mann–Whitney *U* test and unpaired *t*-test.

**Table 3:** Influence of HT on plasma levels of MMP-2, MMP-9, and TIMP-4.

	CAD + HT + DM−	Control	*p* value	CAD + HT + DM−	CAD + HT − DM−	*p* value
MMP-2 (ng/ml)	133.8 ± 23.4	165.7 ± 19.1	**<0.001**	133.8 ± 23.4	134.5 ± 23.2	0.93
MMP-9 (ng/ml)	40.9 ± 23.3	47.2 ± 22.3	0.40	40.9 ± 23.3	47.6 ± 29.4	0.46
TIMP-4 (pg/ml)	1573.1 ± 765.6	1153.2 ± 339.9	**0.038**	1573.1 ± 765.6	1733.0 ± 665.4	0.55

CAD + HT + DM−: group of patients suffering from coronary artery disease and hypertension without diabetes; CAD + HT − DM−: group of patients suffering from coronary artery disease only; MMP-2: matrix metalloproteinase-2; MMP-9: matrix metalloproteinase-9; TIMP-4: tissue inhibitor of metalloproteinases 4. Mean ± SEM. Statistical tests used: Mann–Whitney *U* test and unpaired *t*-test.

**Table 4:** Influence of DM on plasma levels of MMP-2, MMP-9, and TIMP-4.

	CAD + HT + DM +	Control	*p* value	CAD + HT + DM +	CAD + HT + DM−	*p* value
MMP-2 (ng/ml)	175.6 ± 61.5	165.7 ± 19.1	0.81	175.6 ± 61.5	133.8 ± 23.4	**0.0025**
MMP-9 (ng/ml)	50.8 ± 28.4	47.2 ± 22.3	0.67	50.8 ± 28.4	40.9 ± 23.3	0.16
TIMP-4 (pg/ml)	1679.6 ± 784.2	1153.2 ± 339.9	**0.0099**	1679.6 ± 784.2	1573.1 ± 765.6	0.60

CAD + HT + DM + : group of patients suffering from coronary artery disease, hypertension, and diabetes; CAD + HT + DM−: group of patients suffering from coronary artery disease and hypertension without diabetes; MMP-2: matrix metalloproteinase-2; MMP-9: matrix metalloproteinase-9; TIMP-4: tissue inhibitor of metalloproteinases 4. Mean ± SEM. Statistical tests used: Mann–Whitney *U* test and unpaired *t*-test.

**Table 5:** Multiple regression analysis of the influence of independent variables such as HT, DM, and CAD stage on MMP-2, MMP-9, and TIMP-4 concentrations.

*N* = 70	Summary of dependent variable regression: MMP-2, *R* = 0.41825213, *R*^2^ = 0.17493485; corrected *R*^2^ = 0.13743188, F (3.66) = 4.6646, *p* < 0.00512; error standard estimation: 47.404					
	*b* ^*∗*^	Standard error with *b*^*∗*^	*b*	Standard error with *b*	*t* (66)	*p*
W. free			137.9169	23.27753	5.924892	**0.000001**
HT	−0.021563	0.117932	−2.8099	15.36775	−0.182845	0.855480
DM	0.425553	0.118340	43.1472	11.99863	3.596006	**0.000618**
CCS II/CCS III	−0.011262	0.112410	−1.7938	17.90410	−0.100191	0.920496

CCSII/CCSIII: Canadian Cardiovascular Society classification of coronary artery disease clinical status; DM: diabetes; HT: hypertension; MMP-2: matrix metalloproteinase-2; W. free: word free. Statistical test used: ANOVA.

## Data Availability

The study data used to support the findings of this study are not available because these data are confidential.

## Additional Points

*Limitations*. There were only two patients with diabetes in the CAD group without hypertension—this group was not taken into account in the statistical analysis due to the insufficient number of patients. It was difficult to select a group of patients suffering from both CAD and HT, but without DM. Moreover, the influence of DM on the results was observed to be essential in the study group. Furthermore, CAD, HT, and DM overlapped which made it difficult to correspond the obtained results to the other researchers' results. Further study is needed to control the mechanism of MMP/TIMP balance in CAD with comorbidities.
